# Research progress on the mechanism of aging of vascular endothelial cells and the intervention of traditional Chinese medicine: A review

**DOI:** 10.1097/MD.0000000000032248

**Published:** 2022-12-09

**Authors:** Jiang Wen, Caixia Liu, Changqing Deng

**Affiliations:** a College of Integrated Traditional Chinese and Western Medicine, Hunan University of Chinese Medicine, Changsha, Hunan, China; b Key Laboratory of Hunan Province for Integrated Traditional Chinese and Western Medicine on Prevention and Treatment of Cardio-Cerebral Diseases, Hunan University of Chinese Medicine, Changsha, China.

**Keywords:** aging mechanism, cardiovascular disease, cellular senescence, traditional Chinese medicine, vascular endothelial cell, vascular senescence

## Abstract

Vascular senescence is the basic factor of many cardiovascular diseases. Vascular endothelium, as a protective barrier between blood and vascular wall, plays an important role in maintaining the integrity and homeostasis of vascular system. Endothelial cell senescence is an important pathological change of vascular senescence. In recent years, more and more studies have been conducted on vascular endothelial cell senescence, especially on its mechanism. Many research results showed that the mechanism is various, but the systematic elucidation still lacks. Western medicine has little choice in the prevention and treatment of endothelial cell senescence, and the control effect is also limited, while Chinese medicine makes up for the deficiency in this regard. The main mechanisms of vascular endothelial cell aging and the related research progress of traditional Chinese medicine in the prevention and treatment of vascular endothelial aging in recent years were summarized in this paper to provide reference for the research of traditional Chinese medicine in anti-vascular aging and the prevention and treatment of cardiovascular disease.

## 1. Introduction

Aging is 1 of the hotspots in biomedical research, which is accompanied by the development of aging-related diseases. Aging can be considered as a practical disease with aging-derived comorbidities, including cancer or cardiovascular disease. At present, cardiovascular diseases Cardiovasculardiseases, including atherosclerosis, are considered premature aging and are the leading cause of death in developed countries, accounting for 31% of global annual deaths. Aging-related diseases are also increasingly threatening people’s life and health. A large amount of evidence shows that human aging begins with vascular aging and blood cell aging, and vascular aging significantly increases the risk of various cardiovascular diseases. The risk of cardiovascular diseases increases with age, and more than 90% of cardiovascular disease deaths occur over the age of 55.^[1]^ The aging of vascular system is characterized by intimal thickening, arterial stiffness, endothelial dysfunction and chronic vascular inflammation.^[2]^ Vascular endothelial cells are single-layer flat squamous epithelial cells located on the inner surface of blood vessels and anchored to the basal layer of vascular endothelium. In addition to serving as a protective barrier between blood and vascular walls, they are also important cells to maintaining the integrity of the vascular system and homeostasis of the internal environment.^[3]^ Endothelial cell aging is an important sign of vascular aging, and it is also the main cause of cardiovascular diseases such as hypertension and atherosclerosis.^[4]^Therefore, it is of great significance to explore the mechanisms of vascular endothelial cell aging and its intervention measures for the prevention and treatment of cardiovascular diseases.

## 2. The main mechanism of vascular endothelial cell senescence

### 2.1. Oxidative stress

Oxidative stress damage of endothelial cells (EC) is closely related to age-related diseases such as atherosclerosis, diabetes and neurodegenerative diseases. The accumulation of intracellular reactive oxygen species (ROS) is an important cause of aging diseases. ROS accumulation causes oxidative damage to cell membrane and DNA. Oxidative stress is an important factor in promoting vascular endothelial cell senescence when oxidative damage is accumulated and the response and repair ability of the body is unbalanced. ROS is mainly produced in mitochondria and is the metabolite of oxygen molecules in cells. Therefore, the process of oxidative stress in cells is closely related to the integrity of mitochondrial function. Studies have shown that hyperglycemia (HG) can promote mitochondrial dysfunction and induce ROS production, and the resulting ROS can further aggravate mitochondrial damage.^[5,6]^ MiR-34a is a key molecule in HG-induced premature aging of retinal microvascular EC. It participates in mitochondrial dysfunction through many pathways.^[7]^ At the same time, obesity, inflammation, dyslipidemia and other factors can induce ROS production. These factors can lead to vascular injury and promote vascular endothelial aging through ROS-mediated oxidative stress.^[8]^ At present, there are many studies on the prevention and treatment of vascular endothelial cell senescence through antioxidation. Some studies have found that the use of reduced coenzyme Q10 (QH) and vitamin K2 (MK-7) can effectively combat the premature aging of human umbilical vein endothelial cells (HUVECs) induced by cigarette smoke extract. They mainly achieve the anti-senescence effect by increasing the cell vitality of HUVECs and improving their oxidative stress and inflammatory response.^[9]^

### 2.2. Metabolic abnormality

Abnormal metabolism mainly includes abnormal glucose metabolism and abnormal lipid metabolism. Studies have shown that abnormal metabolism is an important factor in inducing vascular endothelial cell senescence, and its main mechanism is that abnormal metabolism induces endothelial cell inflammation and oxidative stress, leading to endothelial cell dysfunction and promoting cell senescence. Studies have shown that under HG condition, the expression level of SIRT3 in HUVECs was decreased, the proportion of cells expressing senescence-associated β-galactosidase was increased, and HG damaged the tube-forming ability of cells.^[10]^ At the same time, HG is the mediator of NLRP3 activation in EC. A variety of hypoglycemic drugs can protect EC and reduce vascular complications of diabetes by inhibiting NLRP3 inflammatory bodies.^[11,12]^ Xu K et al Analyzed RNA-Seq data comprehensively by establishing transcriptomic formulas based on biological knowledge of EC and found that the expression of pro-inflammatory lipid lysophosphatidylinositol was significantly increased in hyperlipidemia. They also found that lysophosphatidylinositols activated the mitochondrial mechanism of HAECs by up-regulating mitochondrial (MitoCarta) gene encoded by 152 nuclear DNA, and activated ROS mechanism of HAECs by up-regulating 18 ROS regulators.^[13]^ Therefore, long-term exposure of vascular endothelium to HG and hyperlipidemia can lead to premature aging of EC.

### 2.3. Chronic inflammatory reaction

Aging EC often at a chronic low-grade inflammatory state, secrete inflammatory cytokines and promote white blood cells into the blood vessels to participate in the occurrence and development of atherosclerosis. Chronic low-grade inflammation, characterized by increased levels of circulating cytokines and infiltration-associated immune inflammation, exacerbates the loss of resilience and increase the risk of disease with age.^[14]^The gene chip analysis of isolated coronary artery after myocardial infarction showed that the gene expressions of TNFα, IL-1β, IL-6, IL-6Rα and IL-17 in young (3 months old) and elderly (25 months old) male rats were significantly increased, which was consistent with age-related pro-inflammatory transformation.^[15]^Aging cells not only damage their own functions, but also affect the surrounding cells by secreting senescence-related secretory phenotype (SASP)-related factors.^[16]^SASP is a marker of aging cells, and inflammatory factors such as TNFα, IL-6 and IL-8 are typical SASP markers, which play key roles in the process of SASP effect.^[17]^ Aging cells and SASP reshape the tissue microenvironment and destroy tissue homeostasis by secreting extracellular matrix and recruiting immune cells.

### 2.4. Activation of renin-angiotensin-aldosterone system

Angiotensin (ANG) is a major active substance from the renin-angiotensin-aldosterone system. It is known that ANG can contract blood vessels and regulate blood pressure, and plays an important role in the pathogenesis of hypertension. Studies have shown that aging-related vascular changes and hypertension have some common pathogenesis at the molecular and cellular level.^[18]^ In recent years, many studies have also confirmed this point from various aspects: ANG can participate in the process of vascular endothelial aging by inducing endothelial dysfunction, endothelial cell apoptosis and promoting oxidative stress. It has been found that Ang Ⅱ promotes endothelial cell senescence by up-regulating the levels of Sodium-glucose cotransporter 1 and Sodium-glucose cotransporter 2 proteins in EC, causing sustained extracellular carbohydrate and sodium-dependent pro-oxidation.^[19]^ Ang Ⅱ can also accelerate vascular senescence by regulating PPARα signal pathway, regulating cellular bypass and transcellular pathway, and increasing the permeability of cerebral vascular EC.^[20]^ Short-term Ang Ⅱ stimulation can promote cell proliferation, migration and angiogenesis, while chronic Ang Ⅱ stimulation inhibits endothelial cell activity, induces apoptosis, and increases the expression of pro-inflammatory cytokines and adhesion molecules, resulting in endothelial senescence and dysfunction.^[21]^

### 2.5. Other

In addition to the above, there are many factors related to the vascular EC senescence. Autophagy is a protective mechanism of the body that can improve oxidative stress injury. In recent years, autophagy has been found to be related to endothelial cell senescence. It is down-regulated in the process of aging, and moderate enhancement of autophagy activity can delay aging. The senescence of EC is related to the decreased expression of several autophagy marker proteins, and autophagy protects arterial EC from injury by reducing oxidative stress and inflammation and increasing the bioavailability of nitric oxide (NO).^[22]^ Ang Ⅱ stimulation can induce HUVECs autophagy, and autophagy activation can improve HUVECs dysfunction induced by Ang Ⅱ, and nicotinamide adenine dinucleotide phosphate oxidase and ROS are involved in this signal transduction process.^[23]^ 17β-estradiol (17β-E2) can promote autophagy by up-regulating SIRT3 gene expression, improve mitochondrial dysfunction induced by H_2_O_2_, thus inhibit H_2_O_2_-induced HUVECs senescence.^[24]^ The change of Ca2 + signal in vascular endothelium is also related to senescence. The activation of calcium-activated potassium channels mainly expressed in the small conductance and the medium conductance of EC can lead to vasodilation, but this function of EC will decrease with aging. Some researchers used potassium calcium channel activator SKA-31 to treat elderly male SD rats, and found that it could improve the endothelium-dependent vasodilation function and the decline of cardiac function caused by aging.^[25]^ In addition, endothelial progenitor cells (EPC) can replace damaged or dead EC to repair vascular endothelium, thus improving vascular endothelial cell senescence. However, with the increase of age, the biological function of EPC decreases. Studies have shown that Nuclear factor erythroid2-related factor 2 (NRF2) can alleviate oxidative stress and inflammatory injury of EPC in elderly mice through NF-κB signal pathway, thus improving endothelial senescence.^[26]^

## 3. Traditional Chinese medicine research on improving vascular endothelial aging

### 3.1. Traditional Chinese medicine compound prescription

#### 3.1.1. Buyang Huanwu Decoction (BYHWD).

Traditional Chinese medicine theory believes that qi deficiency and blood stasis is the main pathogenesis of vascular endothelial aging, and the method of invigorating qi and promoting blood circulation is an important means to delay vascular endothelial aging. BYHWD was founded by Wang Qingren, a famous physician in the Qing Dynasty, and was widely used in aging-related cardiovascular and cerebrovascular diseases. Studies have shown that BYHWD can inhibit mitochondrial dysfunction of HUVECs mediated by ROS and reduce H_2_O_2-_induced endothelial cell apoptosis.^[27]^The compatibility of the main components of BYHWD, Astragali Radix and Angelicae Sinensis Radix, has the protective effect on oxidative damage of HUVECs induced by oxidized low density lipoprotein. The compatibility of these active components can inhibit the production of ROS and promote the expression of cell cycle related proteins, thereby promoting the proliferation of EC and preventing oxidative damage of EC.^[28,29]^

#### 3.1.2. Huoxue Compound.

Huoxue Compound is composed of Huangqi (*Astragali Radix*), Puhuang (*Typhae Pollen*), Jiangxiang (*Dalbergiae Odoriferae Lignum*), Danshen (*Salviae Miltiorrhizae Radix Et Rhizoma*), Cangzhu (*Atractylodis Rhizoma*), Baizhu (*Atractylodis Macrocephalae Rhizoma*) and Zhigancao (*Glycyrrhizae Radix Et Rhizoma Praeparata Cum Melle*). Through clinical and animal experiments, researchers have found that Huoxue Compound can reduce Serum total cholesterol and Triglyceride, which is superior to simvastatin in reducing whole blood, plasma viscosity, fibrinogen and improving symptoms of patient. At the same time, it can enhance or activate the expression of PPARγ gene, increase NO level, inhibit the expression of ICAM-1 and VCAM-1 in vascular wall, protect vascular endothelial integrity, and resist atherosclerosis, thus improving vascular aging.^[30]^

#### 3.1.3. Yiqi Huoxue Yangyin prescription.

Liu Yiqing et al found that Yiqi Huoxue Yangyin prescription could improve the aging performance of heart and aorta in mice induced by D-galactose and delay the development of cardiovascular aging.^[31]^ The mechanism of anti-vascular aging may be related to the decrease of mitochondrial ROS content, the increase of membrane potential and the increase of mitochondrial energy metabolism related complex activity. Yiqi Huoxue Yangyin prescription can delay the aging of heart and aorta by protecting mitochondrial energy metabolism.

### 3.2. Proprietary Chinese medicine

#### 3.2.1. Shuan-Tong-Ling.

Shuan-Tong-Ling is a fermented product of 14 traditional Chinese medicines, which comes from Sanpian decoction, a traditional prescription for the treatment of migraine. It is an empirical prescription for the treatment of vascular encephalopathy. Studies have shown that Shuan- Tong-ling has a protective effect on H_2_O_2_-induced injury of rat brain microvascular EC. The mechanism may be that Shuan- Tong-ling protects brain microvascular EC from oxidative stress through SIRT1/PGC-α and SIRT1/P21 pathway.^[32]^

#### 3.2.2. Naoxintong capsule

Naoxintong capsule is a proprietary Chinese medicine prepared by more than a dozen herbs,such as Huangqi (*Astragali Radix*), Danshen (*Salviae Miltiorrhizae Radix Et Rhizoma*), Chishao (*Paeoniae Radix Rubra*), Chuanxiong (*Chuanxiong Rhizoma*), Taoren (*Persicae Semen*), Honghua (*Carthami Flos*) and so on. It has the effect of invigorating qi and promoting blood circulation. It is often used in the treatment of cerebral infarction and coronary heart disease. Recent studies have found that Naoxintong capsule can improve endothelial cell senescence induced by TNFα, and its anti-aging effect is mainly mediated by regulating the SIRT1 signal pathway.^[33]^

### 3.3. Traditional Chinese medicine monomer

#### 3.3.1. Resveratrol.

Many traditional Chinese medicine components play a role in the prevention and treatment of vascular endothelial aging, and resveratrol is the widely studied and representative 1. Resveratrol was first isolated from the roots of the Chinese herbal medicine Veratrum grandiflorum, which has antibacterial and antioxidant effects. Cheang WS et al established mice endothelial dysfunction model by feeding with high-fat diet, and established the SIRT1 high-expression mice model and PPARδ knockout mice model. It was found that resveratrol could effectively improve endothelial dysfunction through SIRT1 and PPARδ signal pathway, and resveratrol increased the transcriptional activity of PPARδ in EC.^[34]^ Sha W et al also found that resveratrol improved human glycated low-density lipoprotein-induced apoptosis, inflammatory factor secretion and oxidative stress of vascular EC by regulating miR-142-3p and sprouty-related EVH1 domain 2-mediated autophagy.^[35]^

#### 3.3.2. Rhynchophylline (Rhy).

Rhy comes from the traditional Chinese medicine Gouteng (*Uncariae Ramulus Cum Uncis*). It has the effect of Antihypertensive and antithrombotic and is commonly used in the treatment of Cardiovascular and cerebrovascular diseases. Studies have found that Rhy can promote autophagy by activating Adenosine 5‘-monophosphate -activated protein kinase signal pathway, and the enhanced autophagy can delay the senescence of EPC, thus protecting EPC from ANG Ⅱ damage.^[36]^

#### 3.3.3. Puerarin.

Puerarin is extracted from the traditional Chinese medicine Gegen (*Puerariea Lobatae Radix*). It is the main active substance of Gegen (*Puerariea Lobatae Radix*), which has the effects of reducing blood sugar and blood lipid, antioxidant stress, anti-infection and protecting blood vessels. Studies have shown that puerarin can not only inhibit the expression of HUVECs tissue factor induced by oxidative stress by up-regulating PI3K/Akt/eNOS signal pathway,^[37]^ but also reduce H_2_O_2_-induced HUVECs oxidative damage by improving mitochondrial respiratory function.^[38]^

#### 3.3.4. Tanshinone IIA.

Tanshinone ⅡA is 1 of the main active components of Danshen (*Salviae Miltiorrhizae Radix Et Rhizoma*), which has the effects of anti-platelet aggregation and repairing vascular endothelium. Studies have found that tanshinone ⅡA can inhibit the expression of VACM-1 and ICAM-1 in HUVECs induced by TNFα, and improve its inflammatory response by regulating IKK/NF-κB signal pathway.^[39]^At the same time, tanshinone y response by regulating IKK/NF-airing vascular endothelium. Studies have.^[40]^

#### 3.3.5. Corylin.

Corylin, an effective component of traditional Chinese medicine Buguzhi (*Psoraleae Fructus*), has the effects of antioxidation, anti-inflammation and anti-proliferation. Studies have shown that Corylin can reduce atherosclerotic lesions, ROS production and VCAM-1 expression in ApoE knockout mice, and improve vascular cell inflammation, proliferation and migration. The effect is mainly through the activation of ROS/JNK signal pathway.^[41]^

#### 3.3.6. Chlorogenic acid (CGA).

CGA mainly is mainly derived from the traditional Chinese medicine Jinyinhua (*Lonicerae Japonicae Flos*) and Duzhongye (*Eucommiae Folium*), with antihypertensive, antibacterial, antioxidant and other effects. Studies have confirmed that CGA can improve vascular aging and protect HUVECs from oxidative stress injury. Its anti-vascular aging effect may related to the inhibition of cell mitochondrial apoptosis^[42]^ or NRF2/HO-1 signal pathway.^[43]^

#### 3.3.7. Genistein.

Genistein mainly exists in legumes, mainly extracted from traditional Chinese medicine Huaijiao (*Sophorae Fructus*), Shandougen (*Sophorae Tonkinensis Radix Et Rhizoma*),etc., with anti-oxidant, anti-tumor and blood vessels protection and other effects. It can reduce the expression of P16, P21, TXNIP, NLRP3, caspase-1 and caspase-3 in HUVECs induced by H_2_O_2_ and improve the senescence of HUVECs, which is achieved by inhibiting the TXNIP/NLRP3 axis.^[44]^

#### 3.3.8. Monotropein.

Monotropein is extracted from the dried root of Bajitian (*Morindae Officinalis Radix*), which has the effects of anti-inflammatory and antioxidative. Monotropein has a protective effect on vascular EC. It has been found that Monotropein can reduce H_2_O_2_-induced apoptosis, inflammatory reaction and oxidative stress injury of HUVECs by regulating NF-κB/AP-1.^[45]^

#### 3.3.9. Salicin.

Salicin is the main active component of willow bark, which has anti-inflammatory, antipyretic and analgesic effects. It was found that salicyloside could significantly improve the senescence of HUVECs by using TNF-α to induce the senescence of HUVECs and then applying salicyloside to these cells. The action pathway may related to the promotion of NRF2 nuclear translocation by salicyloside, which is different from the mechanism of aspirin.^[46]^

#### 3.3.10. Alismol.

Traditional Chinese medicine Zexie (*Alismatis Rhizoma*) has the effect of diuresis, dampness, turbidity and lipid reduction. Alismol is the main active ingredient of Zexie (*Alismatis Rhizoma*). Its clinical efficacy is to reduce blood lipid and anti-allergy. Studies have found that Alismol A24-acetate can improve the aging of brain microvascular EC by inhibiting the expression of miR-92a-3p.^[47]^

#### 3.3.11. Salidroside.

Salidroside is derived from Hongjingtian (*Rhodiolae Crenulatae Radix Et Rhizoma*), a traditional Chinese medicine of Sedum family. It has been proved to have the effects of improving immunity and protecting cardiovascular system. Studies has found that salidroside can reduce intracellular lipid deposition, inhibit the expression of aging-related molecules, delay the process of EC senescence.^[48]^

#### 3.3.12. 2,3,5,4’-tetrahydroxystilbene-2-o-β-d-glucoside.

As the main effective components of Heshouwu (*Polygoni Multiflori Radix*), 2,3,5,4’-tetrahydroxystilbene-2-o-β-d-glucoside has been known to have the effects of antioxidation, reducing blood lipids, protecting blood vessels. Studies has found that it can enhance the expression of longevity gene Klotho, reduce the number of senescent cells induced by ANG Ⅱ, and improve the antioxidant capacity of the body.^[49]^

#### 3.3.13. Quercetin.

Quercetin is widely found in many plants, with expectorant, antitussive, antihypertensive, lipid-lowering and anti-cancer effects. This component exists in Sangjisheng (*Taxilli Herba*), Kuandonghua (*Farfarae Flos*) and Cebaiye (*Platycladi Cacumen*). Quercetin can reduce the aortic lipid deposition of ApoE^-/-^ mice, the expression of IL-6 and VCAM-1 and increase aortic SIRT1 expression. It can also reduce the expression of HAECs senescence-related β-Gal, the production of ROS, the apoptosis and improve cell morphology. Its function of improving vascular endothelial cell senescence can be confirmed by in vivo and in vitro experiments.^[50]^

#### 3.3.14. Curcumin.

Curcumin is a polyphenolic compound isolated from Jianghuang (*Curcumae Longae Rhizoma*), and has the effects of anti-inflammation, anti-oxidation and anti-cancer. Researchers took postmenopausal women as the research object,and found that after regular intake of curcumin, the systolic blood pressure of the subjects decreased, and the age-related endothelial dysfunction was improved.^[51]^ Other studies have shown that curcumin can improve H_2_O_2_-induced HUVECs premature senility, reduce the positive rate of β-Gal and the expression of aging-related proteins, improve oxidative stress and apoptosis. The possible mechanism is that curcumin reduces oxidative stress injury by activating SIRT1.^[52]^

#### 3.3.15. Icariin.

Icariin, as the main active component of Yinyanghuo (*Epimedii Folium*), has the effects of anti-aging, anti-tumor and regulating immunity. Studies have found that icariin can increase the expression of SIRT6 in mouse heart tissue and thoracic aorta, and inhibit the expression of NF-κB and its downstream genes TNFα, ICAM-1, IL-2 and IL-6 in these 2 tissues. It is confirmed that icariin can improve cardiac inflammation and endothelial senescence through NF-κB pathway.^[53]^

#### 3.3.16. Epifriedelanol.

Elm is a traditional Chinese medicine. Its fruits, bark, roots and leaves can be used as medicine. Epifriedelanol is mainly extracted from the root bark of elm and has the effects of anti-inflammation, anti-oxidation and anti-cancer. Studies have shown that it can inhibit the replicative senescence of HUVECs and the premature senescence of HUVECs induced by doxorubicin, which may related to the antioxidant activity of epifriedelanol.^[54]^

### 3.4. Traditional Chinese medicine extracts

#### 3.4.1. Crataegus extract.

Crataegus Extract is derived from the traditional Chinese medicine Shanzha (*Crataegi Fructus*),and has the effects of antihypertensive, lipid-lowering, antibacterial, anti-inflammatory. Some studies have carried out in vivo and in vitro experiments on the coronary artery EC of rat, and found that long-term intake of Crataegus special extract WS1442 can improve aging-related endothelial dysfunction, which may be achieved by inhibiting oxidative stress and overexpression of COX-1 and COX-2.^[55,56]^

#### 3.4.2. Ginseng-Sanqi-Chuanxiong (GSC) extract.

GSC is extracted from the proportionate combination of 3 traditional Chinese medicine of Renshen (*Ginseng Radix Et Rhizoma*), Sanqi (*Notoginseng Radix Et Rhizoma*) and Chuanxiong (*Chuanxiong Rhizoma*).Studies have found that GSC can not only regulate mitotic regulation through adenosine 5‘-monophosphate -activated protein kinase pathway to prevent high glucose and palmitate (HG/PA)-induced endothelial senescence^[57]^，but also improve the senescence of microvascular EC by regulating heat shock protein 27 to down-regulate F-actin expression.^[58]^The active component of Ginsenoside Rb1 is relative more studied, which has a significant inhibitory effect on vascular endothelial cell senescence induced by oxidative stress, but its action pathway is diverse^[59,60]^，and Ginsenoside Rb2 has a similar effect.^[61]^

#### 3.4.3. Aralia elata (Miq.) seem extract.

Aralia elata (Miq.) Seem is a kind of Araliaceae plant from northeast China, with the effects of invigorating qi for tranquilization,nourishing kidney and activating blood.The Aralia elata (Miq.) Seem extract can improve the cell cycle arrest of senescent HUVECs induced by high glucose, increase the expressions of SIRT1 and eNOS, and improve endothelial cell senescence by regulating AKT/eNOS signal pathway.^[62]^

### 3.5. Other natural products

There are a variety of substances with anti-aging effects of vascular EC. Among them, natural products from traditional Chinese medicine have been studied most, but there are also natural products from nontraditional Chinese medicine have similar effects.

#### 3.5.1. Soybean extract.

Soybean isoflavones that extracted from legumes have been found to improve endothelial cell function, resist oxidative stress through estrogen receptors, delay cell senescence, and delay the progression of atherosclerosis to some extent.^[63]^Soybean seed coat polyphenols has also been found to promote NO production in rat aorta and improve vascular function. The mechanism may be that GLP-1 secreted by polyphenols substances contained in it activates eNOS in vascular EC.^[64]^

#### 3.5.2. Anthocyanins.

Anthocyanins widely found in the cytosol of flowers, fruits, stems, leaves and roots of plants, have been found to increase NO bioavailability by regulating ROS formation and reducing eNOS uncoupling, thus improving the senescence of EC.^[65]^ Sour Cherry extract has also been proved to have antioxidant, anti-inflammatory and vasodilating effects, and can improve vascular dysfunction caused by high glucose, which is also due to its rich in anthocyanins.^[66]^

#### 3.5.3. N-butanol extracts of morinda citrifolia.

Morinda citrifolia, commonly known as the noni berry, is a plant of Morinda officinalis of Rubiaceae. It has anti-inflammatory, anti-cancer, antioxidant and other effects, and has high medicinal value. It has been found that the N-butanol extract of Morinda citrifolia can inhibit the inflammatory response of HUVECs induced by AGE, and this effect is achieved by inhibiting the AGE-RAGE axis and blocking the interaction between AGE and RAGE.^[67]^This may be a new way to prevent and control cardiovascular disease.

#### 3.5.4. Cacao polyphenols.

Cacao Polyphenols are extracted from cacao kernels of Sterculiaceae, and have antioxidant effects. The researchers found that Cacao Polyphenols could improve the anti-platelet aggregation of aging EC, increase the secretion of NO and regulate blood lipids. It is confirmed that Cacao Polyphenols can improve the senescence of vascular EC and protect cardiovascular system through the above ways.^[68]^

#### 3.5.5. Red wine extract.

Red wine is a common drink in daily life, which has the effects of anti-oxidation, anti-aging and cardiovascular protection. There is evidence that red wine extract can reduce the DNA damage of EC and inhibit endothelial cell senescence induced by oxidative stress. This is mainly achieved by up-regulating eNOS expressions and NO production in EC, clearing ROS and improving oxidative stress injury.^[69]^

#### 3.5.6. White tip silver needle flavonoids.

White Tip Silver Needle, produced in Fujian, China, is a kind of Slightly fermented white tea with antioxidant and anti-inflammatory effects. White Tip Silver Needle Flavonoids can reduce liver, kidney and lung injury in aging mice induced by high glucose, and reduce the expression of inflammatory and oxidative stress related genes in mice.^[70]^

#### 3.5.7. Pummelo fruit extract.

Pummelo is a common citrus fruit. Its fruit extract has been found to promote the migration and proliferation of HUVECs, reduce the level of ROS in senescent cells, increase the expression of eNOS gene, delay HUVECs senescence and reduce the risk of cardiovascular disease.^[71]^

#### 3.5.8. Other.

In addition to the above components, there are many other natural products also have the effect of delaying endothelial aging, but most of them improve aging by inhibiting oxidative stress in the human body.For example, Spa resorts mud extract can reduce VCAM-1 expression,increase the expressions of SIRT1, FOXO3 and SOD1 in EC, which shows strong anti-inflammatory, antioxidant and anti-aging effects.^[72]^ A natural extract from stem bark of Mangifera indica L. extract (Vimang) can improve the serum antioxidant capacity of the elderly and reduce the expression of age-related oxidative stress-related factors.^[73]^

## 4. Conclusion

The incidence of cardiovascular disease is increasing, and its incidence tends to be younger, threatening people’s health, so the study of prevention and treatment of cardiovascular disease is becoming more and more important. And it is necessary to make an in-depth study on its pathogenesis to prevent and treat cardiovascular diseases. There are many pathways can mediate vascular endothelial cell senescence, each of which has its own independence but also affects each other and is interrelated through oxidative stress. However, there is a lack of comprehensive and systematic exposition on the mechanism of vascular endothelial senescence. Therefore, the future research on mechanisms of vascular endothelial aging can focus on the interaction of various pathways, so as to have a more comprehensive understanding. In addition, current studies have shown that the prevention and treatment of aging-related cardiovascular diseases is mainly to prevent their exposure to cardiovascular risk factors. This strategy is effective in reducing mortality associated with these diseases, but its effectiveness seems to have reached a limit, as evidenced by the high incidence of these diseases. Expanding the research on the mechanism of regulating endothelial aging can develop new treatments. According to the above, traditional Chinese medicine plays an important role in the fight against vascular endothelial cell aging, showing significant advantages, and has broad research prospect, which provides a new direction and ideas for the prevention and treatment of cardiovascular diseases. Therefore, a comprehensive and in-depth exploration of vascular endothelial mechanism, and the use of the advantages of traditional Chinese medicine to study its new prevention and treatment measures are the follow-up problem that needs to be further solved.

## Acknowledgments

This work was funded by grants #81874406, #82104650 from the National Natural Science Foundation of China, grant #2022JJ30431 from the Hunan Natural Science Foundation, grant #2020XJJJ015 from Research Fund Project of Hunan University of Chinese Medicine, and #2021ZXYJH05 from major Project of Integrated Chinese and Western Medicine (First-Class Discipline) of the Hunan University of Chinese Medicine.

## Author Contributions

**Conceptualization:** Jiang Wen.

**Data curation:** Jiang Wen.

**Editing:** Jiang Wen.

**Reviewing and editing:** Caixia Liu, Changqing Deng.

**Funding acquisition:** Caixia Liu, Changqing Deng.
